# The Effect of Dopaminergic Therapies in Parkinson’s Disease on Non-Motor Symptoms

**DOI:** 10.3390/ijms262411996

**Published:** 2025-12-12

**Authors:** Monika Jampolska, Katarzyna Kaczyńska

**Affiliations:** Department of Respiration Physiology, Mossakowski Medical Research Institute, Polish Academy of Sciences, Pawińskiego 5, 02-106 Warsaw, Poland; mjampolska@imdik.pan.pl

**Keywords:** Parkinson’s disease, non-motor symptoms, autonomic dysfunction, sleep-related disorders, neuropsychiatric disorders, pain, cognitive impairment, dopaminergic therapy

## Abstract

Parkinson’s disease (PD) is a neurodegenerative disorder due to damage of the nigrostriatal pathway and consequent dopamine (DA) deficiency. The main classic symptoms are those related to motor disturbances, such as postural instability, resting tremor, bradykinesia, and muscle rigidity. Other symptoms, such as non-motor symptoms, do not attract the attention of clinicians and are often overlooked, remaining undiagnosed and untreated, even though they can significantly impair quality of life of PD patients. Dopaminergic therapy is primarily aimed at treating motor symptoms, although it can bring measurable benefits for various non-motor problems. This narrative review analyzes the scientific literature and describes the most recent information on the impact of dopaminergic therapy on non-motor symptoms in PD; both its beneficial and undesirable effects. We discuss evidence that non-motor symptoms such as cardiovascular dysfunction, thermoregulatory issues, dysphagia and drooling, urinary symptoms, pain, neuropsychiatric symptoms, and sleep disorders, including obstructive sleep apnea, can be effectively treated with various dopaminergic strategies, while noting the contraindications and adverse effects of these therapies.

## 1. Introduction

Parkinson’s disease (PD) is a neurodegenerative disorder attributed to damage to the nigrostriatal pathway, involving the loss of brain cells in the substantia nigra pars compacta (SNpc) responsible for dopamine (DA) production. Dopamine produced in this part of the central nervous system (CNS) and released in the striatum is vital for controlling body movements and coordination [1,2,3]. The main classic motor symptoms, such as postural instability, resting tremor, bradykinesia, and muscle rigidity [4], are well recognized and are the primary focus of clinicians. The dopaminergic nigrostriatal pathway suffers the most significant damage, producing the classic motor symptoms, while the other dopaminergic pathways, mesolimbic, mesocortical, and tuberoinfundibular, are affected to a lesser extent, but still contribute to the development of non-motor symptoms [5,6,7,8].

Non-motor symptoms do not attract the attention of clinicians and are often overlooked, remaining undiagnosed and untreated, although they can significantly impair quality of life. Patients themselves often do not report them either, as they do not associate them with the consequences of Parkinson’s disease. It should be noted that most, if not all, of non-motor symptoms are not specific to Parkinson’s disease. A set of non-specific prodromal symptoms, such as anosmia, constipation, urinary problems, and hypotension, could theoretically become a panel of peripheral biomarkers. However, any attempts to detect prodromal Parkinson’s disease based on these non-specific symptoms or the overuse of PET scans may lead to problems associated with overdiagnosis [9]. The most common non-motor problems observed in PD are: autonomic symptoms, sleep dysfunction, breathing problems, pain, and neuropsychiatric symptoms including depression, apathy, anxiety, and cognitive dysfunction [6,8].

Since the progressive loss of dopamine in the brain in PD causes disintegration of the functioning of individual brain areas related to motor and non-motor symptoms, the gold standard for treating the disease is the dopamine precursor, levodopa [10]. Given the short half-life, adjunctive therapies, including dopamine metabolism inhibitors and dopamine receptor agonists, have been established to prolong dopaminergic activity and maintain therapeutic benefits. Dopaminergic therapy is primarily aimed at treating motor symptoms, but it can play a multidimensional role in the functioning of other areas of the CNS outside the nigrostriatal pathway and modify non-motor symptoms. Relatively little attention is paid to the latter and their treatment.

The aim of this narrative review is to analyze the scientific literature and describe information on the impact of dopaminergic therapy, such as its beneficial effects and side effects on non-motor symptoms in Parkinson’s disease. This will be an update of the information since the detailed review on this topic by Chaudhuri and Schapira, written in 2009 [6], with particular emphasis on the most recent literature based on human studies and symptoms such as cardiovascular dysfunction, thermoregulatory problems, dysphagia and drooling, urinary symptoms, pain, neuropsychiatric symptoms, and sleep-related disorders. In our review article, we have limited ourselves to those non-motor symptoms that have been the subject of new, original reports in recent years. We also took into account, to a considerable extent, respiratory disorders and the therapeutic effect of amantadine, which are usually overlooked in review articles.

## 2. Current Dopaminergic Therapies for Parkinson’s Disease

### 2.1. Levodopa

**Figure 1:**
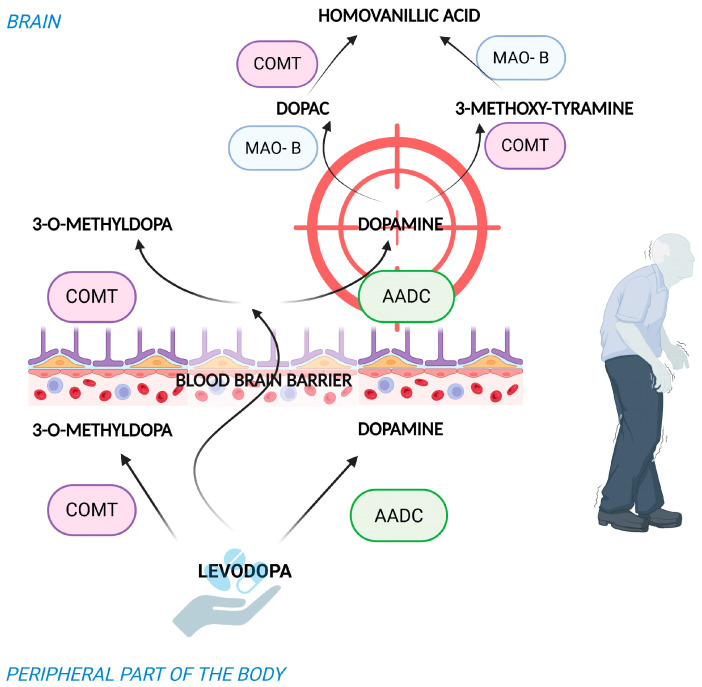
Metabolic pathway of dopamine synthesis after levodopa (L-DOPA) treatment and degradation in the brain by enzymes MAO-B and COMT to its main inactive metabolite, homovanillic acid.

**Figure 2:**
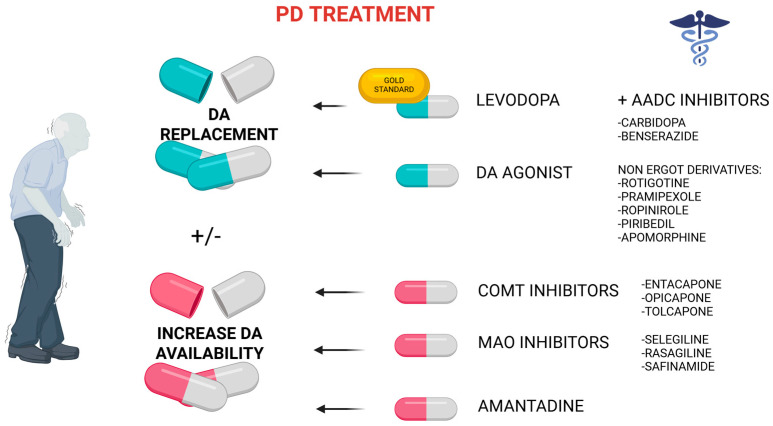
The most popular dopaminergic medicines for Parkinson’s disease and their mechanism of action.

Dopamine (DA) itself is unable to effectively cross the blood–brain barrier (BBB), thus it cannot be replenished in the brain by peripheral administration. Therefore, therapeutic strategy involves its precursor, levodopa (L-DOPA), which easily penetrates the BBB and, in the brain, is converted into dopamine (Figure 1), effectively increasing its level in the striatum, which helps to reduce or control the symptoms of Parkinson’s disease. Levodopa is the golden standard in the treatment of motoric symptoms of PD, but has a relatively short plasma half-life of approximately 90 min, leading to fluctuations in its blood concentration, which in advanced stages of the disease contribute to variations in clinical response, most notably the wearing-off phenomenon [10]. The dose of the drug is increased gradually and adjusted individually, depending on the severity of the symptoms. The clinical effect of levodopa is usually noticeable quickly and persists for several hours, especially in the early stages of the disease. Levodopa is often combined with a peripherally acting levodopa decarboxylase inhibitors such as benserazide and carbidopa (Figure 2), which do not cross the BBB and selectively inhibit the peripheral conversion of levodopa to dopamine, resulting in a reduction in its adverse effects [11]. With the progression of the disease, however, the effect of the drug usually wears off after a shorter period of time requiring an increase in the frequency of dosing. Long-term use is associated with significant side effects, including motor complications such as dyskinesia and severe ‘on-off’ motor fluctuations [12].

### 2.2. Monoamine Oxidase B (MAO-B) and Catechol-O-Methyl Transferase (COMT) Inhibitors

The mechanism of action of other drugs used to treat PD is based on inhibiting enzymes involved in dopamine metabolism, thereby maintaining endogenous dopamine levels. Inhibiting the activity of the MAO-B enzyme, responsible for the breakdown of dopamine in the brain (Figure 1), increases dopaminergic activity in the striatum and helps to control motor symptoms of PD. It can be used as initial therapy in the early stages of the disease, and later in combination with levodopa [10,13]. MAO-B inhibitor adverse effects range from nausea, headaches, insomnia, and dizziness to, in uncommon cases, serotonin syndrome or hypertension [14]. COMT is another enzyme involved in the degradation of dopamine, so applying its inhibitors blocks the degradation or modification of dopamine, improving its effectiveness and duration of action (Figure 1). They are mainly used as supportive therapy for levodopa, prolonging its time of action and helping the control of motor symptoms, especially reducing the duration of the ‘off’ phase over standard combinations of levodopa and a DOPA decarboxylase inhibitor. They are commonly used in patients who present with ‘wearing off’ of the dose at the end of the effect of levodopa [12]. The most commonly observed adverse effect associated with COMT inhibitors is dyskinesia. Less commonly observed are diarrhea and liver toxicity especially with tolcapone [15].

### 2.3. Dopamine Agonists

Another method of treating and activating the dopaminergic system is the use of agonists of dopaminergic receptors, which bind to the receptor and trigger a biological response, mimicking the action of the natural compound or enhancing that response (Figure 2). While dopamine agonists may be less effective than levodopa in controlling the motor symptoms of PD, they may be helpful in patients with mild symptoms, in those who cannot tolerate levodopa, or as an add-on to levodopa therapy [12]. In addition, they have a longer half-life than levodopa and are less likely to cause motor complications after early treatment initiation [16]. Dopamine agonists such as cabergoline, pramipexole, ropinirole, and rotigotine have been reported to be potent in treating Parkinson’s disease, both as monotherapy and in combination with levodopa [17]. They are recommended to counteract the wearing-off phenomenon in the early stages of treatment and in advanced stages of the disease [10].

Among the common adverse effects associated with DA agonists treatment are constipation, nausea, headaches, and excessive daytime sleepiness. In addition to them, hallucinations, impulse control disorders, somnolence, peripheral edema, valvular heart disease, fibrosis, and heart failure have been observed [18]. Of note, dopamine agonists, especially those acting on the D_1_ receptor, may also affect intraocular pressure, and although few studies have been conducted, they show that PD patients are at increased risk of developing glaucoma [19]. Non-ergoline DA (pramipexole, ropinirole, rotigotine, pribedil, apomorphine) have a better safety profile in contrary to ergot-derived (bromocriptine, pergolide, carbegoline, lisurine), which are associated with cardiac valve regurgitations and fibrotic changes [18].

### 2.4. Amantadine

Another activator of the dopaminergic system working by increasing dopamine levels in the brain is amantadine, which amplifies dopamine transmission through presynaptic and postsynaptic mechanisms by enhancing biosynthesis, turnover, uptake, and synaptic release of dopamine from striatal dopaminergic neuron terminals and increasing L-amino acid decarboxylase activity in the striatum, and thus dopamine synthesis (Figure 2) [20]. It was discovered by chance that this well-known antiviral drug for treating flu has a significant effect on the accumulation and release of dopamine in the CNS, which may help to reduce involuntary movements. It can be used as monotherapy or as adjunctive therapy to reduce levodopa doses. Amantadine is used in Parkinson’s disease basically to ease motor parkinsonian symptoms like tremors, stiffness, and bradykinesia, as well as dyskinesia, which occurs after taking levodopa [21]. The amantadine anti-dyskinetic effect has been attributed to its central anti-glutamatergic action [20]. Amantadine is considered to have a mild adverse effect profile. The main adverse effects of amantadine comprise orthostatic hypotension, fainting, dizziness, delusions, hallucinations, falls, dry mouth, and constipation [22].

## 3. Impact of Dopaminergic Therapies in Parkinson’s Disease on the Non-Motor Symptoms

### 3.1. Autonomic Dysfunction in Parkinson’s Disease

#### 3.1.1. Cardiovascular Dysfunctions

Most dysautonomic symptoms arise as a result of changes in the peripheral nerves of the autonomic nervous system. In PD, a decrease in parasympathetic activity is generally observed, accompanied by an increase in sympathetic activity leading to imbalance between the two tonicities. Cardiovascular dysautonomia and less frequently structural cardiac diseases manifest as the most common cardiovascular impairments in PD patients, such as orthostatic hypotension, heart rate variability, changes in cardiogram parameters, and baroreflex dysfunction, which worsen as the disease progresses [23,24]. The development of neurogenic orthostatic hypotension (OH) in PD is most often due to a combination of preganglionic (baroreflex failure) and postganglionic (sympathetic denervation) changes [25,26].

In general, dopaminergic antiparkinsonian drugs that increase dopaminergic neurotransmission do not have a beneficial effect on cardiovascular problems. Dopamine is a catecholamine that raises blood pressure and increases the force of the heart’s contraction, that is used as a drug in life-threatening situations. In addition to the brain, it is also found in the heart, blood vessels, and other organs. Elevated dopamine levels can increase heart rate and the risk of serious arrhythmias, and may therefore be dangerous for PD patients with cardiovascular problems. Consequently, oral levodopa supplementation in combination with benserazide causes a significant decrease in mean arterial pressure, cardiac output and cardiac contractility indices [27]. The authors suggested that hypotension was mainly caused by a negative inotropic mechanism rather than peripheral vasodilation. However, the study does not clarify whether this is the result of peripheral action at the heart level or central inhibition of the sympathetic nervous system. Other cardiovascular symptoms in PD patients treated with levodopa include increased aortic stiffness and impaired diastolic function compared to healthy individuals [28]. Since the number of patients in this cross-sectional study was relatively small (n = 65 patients with PD) and only echocardiographic measurements were used for the study, further studies using a more advanced method of assessing aortic elasticity parameters are needed to confirm this finding. However, the most commonly observed cardiovascular dysfunction resulting from levodopa treatment was the onset and aggravation of orthostatic hypotension [29,30].

##### Orthostatic Hypotension (OH)

A large study with a cohort of 490 PD patients who underwent an acute levodopa challenge test showed sudden OH in over 50% of the subjects. PD patients with a better response to levodopa were more susceptible to its hypotensive effects. Among all participants, 33% of patients had OH even before levodopa treatment. Therefore, it is recommended that, in particular, individuals who have started or increased their dose of levodopa should be monitored for hemodynamic changes in order to avoid OH [29,30].

Smaller cohort studies (83 patients) have indicated that doses of 250 mg or more of levodopa/benserazide can significantly lower blood pressure and cause orthostatic effects in elderly patients with early- to mid-stage Parkinson’s disease [29,30]. A further study of 164 patients with PD showed that those with autonomic cardiovascular insufficiency were more prone to levodopa-induced OH. [31]. Levodopa with carbidopa administered as an enteral gel also caused OH [32,33,34] and, in rare cases, congestive cardiac failure and atrial fibrillation [34].

It is well-known that levodopa, a dopamine precursor, causes peripheral vasodilation by activating D_1_ dopamine receptors in the smooth muscles of blood vessels and inhibiting sympathetic nerve activity, thereby reducing peripheral vascular resistance [35]. Carbidopa, on the other hand, contributes to the formation of OH by inhibiting the action of L-amino acid decarboxylase, an enzyme responsible for the decarboxylation of dihydroxyphenylserine to norepinephrine (NE). This hinders NE production, which can lead to vasodilation. The role of carbidopa was confirmed by a case-based study showing that reducing its dose, in particular, diminished the severity of orthostatic hypotension [36].

There is no information on the effect of MAO-B inhibitors on PD cardiovascular dysfunction, however according to the European label, the use of selegiline in combination with levodopa has a number of contraindications limiting its clinical use, such as hypertension, tachycardia, cardiac arrhythmia, and severe coronary heart disease [37].

When it comes to the cardiovascular side effects of dopaminergic agonists, one of the most commonly referred to is orthostatic hypotension, and the most serious is heart valve fibrosis caused by ergoline compounds. For this reason, one of them, pergolide, was withdrawn by the FDA in the US in 2007. Among serious adverse events, an increased risk of heart failure in PD patients as a result of pramipexole and cabergoline treatment is cautiously suggested [38].

Amantadine, a dopaminergic transmission activator, should also be used with caution in PD patients who experience orthostatic hypotension, as OH is one of its side effects [22].

#### 3.1.2. Thermoregulatory Changes

Thermoregulatory dysfunction is a known but rather rarely reported phenomenon in PD. Dopamine plays a key role in regulating brain temperature, so its deficiency in the brain affected by Parkinson’s disease may influence the body’s thermoregulation. In the brain’s temperature control center, hypothalamus, dopamine excites warm-sensitive neurons and inhibits cold-sensitive neurons, which is a key part of maintaining normal temperature [39,40]. The most commonly observed thermoregulatory disorder is spontaneous and recurrent hypothermia, which can sometimes be severe or life-threatening [41,42,43]. According to Renga et al. [42] hypothalamic dysfunction caused by synucleinopathy, resulting in temperature regulation disorders, may be responsible for episodes of hypothermia. Most cases of hypothermia were accompanied by impaired consciousness and worsening Parkinson’s symptoms. Interestingly, many PD patients experienced bradykinesia shortly before the onset of hypothermia [44]. Levodopa with carbidopa has a positive effect on stabilizing body temperature in patients with PD and accompanying hypothermia by increasing dopamine levels [42]. However, it is of note that levodopa rapid withdrawal may develop acute hyperthermia [26,45].

Additionally, it should be borne in mind that long-term dopaminergic therapy may cause dyskinesia-hyperpyrexia syndrome (DHS), a rare movement disorder emergency associated with Parkinson’s disease, consisting of severe continuous dyskinesias associated with rhabdomyolysis, subsequent alteration of the mental state, and hyperthermia, where the main treatment is to limit the use of dopaminergic drugs [46,47].

Since dopamine is involved in the hypothalamic synapses that control the thermoregulatory responses necessary for heat dissipation [39], PD patients with deficiencies of this catecholamine may experience hypo- or anhidrosis, as well as hyperhidrosis [48]. These symptoms may indicate impaired thermoregulatory function of the hypothalamic–medullary–sympathetic axis or of the sweat glands themselves. Hypohidrosis and anhidrosis are rarely observed unless they reach an extreme form, in which case the resulting heat retention can be life-threatening [49]. Hyperhidrosis, which is significantly correlated with drug withdrawal and dyskinesia in patients with PD [48,50,51], may be the consequence of inadequate central dopamine stimulation [52]. Since profuse sweating is considered part of the spectrum of off-period levodopa associated fluctuations, it is not surprising that therapy with levodopa [53] or agonists such as pergolide [54] or apomorphine [55] is effective in alleviating hyperhidrosis, especially during the ‘off’ period when drug concentration is low [53,54]

#### 3.1.3. Dysphagia and Drooling

Swallowing disorders, or dysphagia, are a very common symptom of Parkinson’s disease, affecting approximately 40–80% of people worldwide at all stages of the disease. Although dysphagia worsens over time and becomes more prominent in the later stages, early symptoms may appear in the prodromal and early stages [56]. Another problem associated with dysphagia is excessive drooling in PD patients, resulting more from ineffective removal of saliva from the mouth due to reduced spontaneous swallowing frequency than from hypersalivation [57].

Dysphagia most often responds to treatment with levodopa, potentially by improving motor symptoms such as rigidity and slowness of movement [58]. Better motor control may lead to more effective swallowing movements, reducing dysphagia and improving clearance of saliva from the oral cavity. Overall, the beneficial effects of treatment with levodopa or dopaminergic agonists arise from improving muscle function during swallowing and reaching the ON state in patients with PD [59,60]. Among dopaminergic agonists, non-ergoline rotigotine was shown to be effective in alleviating swallowing difficulties [61,62]. Amantadine was also reported to improve swallowing ability and reduce drooling after a two-week trial in elderly patients with and without PD [63].

#### 3.1.4. Urinary Symptoms

Urinary tract dysfunctions, such as urgent need to urinate, frequent urination, nocturnal urination, and incontinence are common in patients with PD [64]. In a healthy brain, the basal ganglia exert an inhibitory effect on the brainstem micturition centers in the pons. In Parkinson’s disease, however, neurodegenerative changes in the basal ganglia and weakened dopaminergic function lead to the removal of inhibition of the urination reflex, resulting in detrusor muscle overactivity and urinary urgency [65].

In general, therapy involving dopaminergic system activation is considered to be beneficial in PD urinary symptoms [66]. Administration of extended-release levodopa before bedtime alleviated nocturnal urination in patients with Parkinson’s disease [67]. In their earlier studies, the authors noted that the acute effect of levodopa on bladder function exacerbated urinary urgency, while the long-term effect was beneficial, relieving detrusor overactivity in patients with mild PD [68]. MAO-B inhibitor rasagiline significantly improved bladder dysfunction in patients with mild PD by increasing bladder capacity, improving first desire to void and reducing residual urine volume, most likely due to an increase in dopamine concentration in the synapses at the central level [69]. The use of safinamide, another MAO-B inhibitor, as add-on therapy improved urinary function such as urinary urgency, urinary incontinence, frequent urination, and nocturia [70,71,72].

Amantadine, enhancing the release and inhibiting the reuptake of dopamine, at a daily dose of 150 mg, has beneficial effects on lower urinary tract symptoms and nocturnal polyuria after one month of therapy [73].

Among dopaminergic agonists, apomorphine administered chronically and subcutaneously in advanced PD was reported to alleviate nocturia and urinary urgency [55]. Also another agonist, rotigotine, used for three months in monotherapy, improved bladder function in PD patients by increasing bladder capacity and reducing symptoms of irritation [74], which, as authors suggested, may have resulted from its balanced agonism towards D_1_ and D_2_ receptors, and notably from stimulation of D_1_ receptors in the anterior cingulate cortex and insula, well-known areas engaged in bladder inhibitory functions.

### 3.2. Sleep-Related Disorders in Parkinson’s Disease

PD patients who have not previously been treated with medication exhibit excessive daytime sleepiness (EDS) and nighttime sleep disturbances, such as significantly reduced sleep efficiency, prolonged sleep onset latency and reduced REM sleep (rapid eye movement phase) compared to healthy individuals, suggesting that the neurodegenerative process in PD contributes to changes in sleep architecture [75]. In addition to insomnia and REM behavior disorder (RBD), among the sleep disorders observed in PD are restless legs syndrome (RLS) and sleep-disordered breathing (SDB). One of the most recent polysomnographic studies on 162 patients with early PD showed sleep disorders in 71% of cases, with their number increasing along with the duration of the disease and dysautonomia [76]. The most common disorder was insomnia (41%), followed by pronounced RBD (25%), EDS (25%), and RLS (16%) [76]. Sleep disorders are present in the prodromal and early stages of Parkinson’s disease, but intensify as the disease progresses [77]. Reasons for sleep disturbances in Parkinson’s disease are multifactorial, but the leading mechanisms responsible are degeneration of the thalamocortical pathways and impairment of neurotransmitter systems involved in sleep regulation, such as the dopaminergic system in the ventral tegmental area and substantia nigra, and the hypocretin system in the hypothalamus [78]. In addition to the obvious impact of the disease on the areas of the brain that regulate sleep and neurotransmitters, non-motor symptoms such as depression, pain, and nocturia can also affect sleep and its quality [79]. What is more, medications used exclusively to treat motor symptoms may also affect sleep disorders in Parkinson’s disease. Dopaminergic drugs have a dose-dependent effect on sleep. It is believed that in small doses these drugs induce slow-wave and REM sleep and cause drowsiness via D_2_ autoreceptors, while in large doses they attenuate slow-wave and REM sleep and produce wakefulness [6]. Generally, levodopa and dopaminergic drugs may have variable effects, beneficial or adverse, depending on the dose, route of delivery, and differential effects on various dopamine receptors.

Since there are already several reviews dealing exclusively with sleep disorders in PD and the effects of treatment with dopaminergic activators [77,80,81,82,83,84] in our review we limited ourselves to more recent publications and addressed the overlooked effect of amantadine and the topic of sleep-related breathing disorders.

#### 3.2.1. Night-Time Sleep Disturbances and Excessive Daytime Sleepiness (EDS)

It has been demonstrated that intrajejunal infusion of a gel containing levodopa/carbidopa for an average of 15–16 h per day, being an effective treatment for complex motor symptoms in people with advanced PD, also has a long-term beneficial effect on sleep parameters and its quality [85]. Other extended-release levodopa/carbidopa preparations taken at bedtime in combination with entacapone also provided better control of nocturnal motor symptoms and more continuous sleep in patients with advanced PD and motor fluctuations [86]. Yet, studies comparing three PD cohorts, using the Parkinson’s Disease Sleep Scale (PDSS-2) in two cohorts and a question on nocturnal immobility in one cohort, showed that patients taking high doses of extended-release levodopa alone experienced poorer sleep quality and increased nocturnal akinesia [87]. The study had its limitations. A discrepancy was observed between subjective sleep quality and objectively measured sleep mobility, and the results were obtained from cross-sectional studies, so the observations do not allow conclusions to be drawn about causality. Other factors affecting quality of life, such as depression or medication use, were not taken into account. Nevertheless, research findings indicated that levodopa alone, when used in high doses, may not be suitable for improving sleep quality. This is consistent with later studies showing that adjunctive therapy with levodopa/DOPA decarboxylase inhibitors in the form of opicapon, a COMT inhibitor, improves the quantity and quality of sleep in patients with motor fluctuations at the end of the dose [88,89].

Although DA agonists are associated with excessive daytime sleepiness, their add-on use may improve overall sleep quality in patients with advanced, fluctuating PD, particularly by alleviating motor symptoms at night. According to a retrospective study evaluating a large group of patients with advanced PD, comparing add-on treatment with different DA agonists, rotigotine and pramipexole exhibited a lower risk of sleep disturbances, while no significant effect was observed with ropinirole [90]. A recent study confirmed that rotigotine improved sleep quality in patients who had not previously been treated with DA agonists and also had a positive effect on daytime sleepiness, which may have been due to improved motor function [91]. However, it should be noted that dopamine agonists with selectivity for the dopamine D_3_ receptor, i.e., pramipexole and ropinirole, have been reported as a potential risk factor for excessive daytime sleepiness and sleep attacks, especially in the Park’s sleep subtype, a specific Parkinson’s disease cluster, typically characterized by sleepiness of varying severity, with or without accompanying insomnia [92]. In contrast, apomorphine, a D_1_ and D_2_ receptor agonist, administered by subcutaneous infusion exclusively at night, reduced sleep disturbances and had no effect on daytime sleepiness [93,94]. Beneficial effect of MAO-B inhibitors on sleep disturbances has been proved for rasagiline [95] and safinamide [96]. Safinamide not only improved sleep quality and duration but also reduced excessive daytime sleepiness [97]. The latter was significantly worsened after introduction of COMT inhibitor entacapone [98].

Extended-release amantadine capsules approved for the treatment of dyskinesias and as an adjunct to levodopa in episodes of ‘off’, have been shown to have a beneficial effect on daytime sleepiness in PD patients with dyskinesia [99].

#### 3.2.2. Sleep-Disordered Breathing (SDB)—Obstructive Sleep Apnea (OSA)

One of the non-motor symptoms of PD is sleep-disordered breathing (SDB), specifically obstructive sleep apnea (OSA). OSA is a common sleep disorder characterized by snoring and repeated collapse of the upper airway, leading to arrest of breathing or shallow ventilation, resulting in fragmented nighttime sleep followed by daytime sleepiness and fatigue. Significant nocturnal hypoxemia and blood pressure spikes associated with OSA cause chronic inflammation, oxidative stress, and may exacerbate neurodegenerative changes [100]. The prevalence of OSA in PD is still a matter of debate, as there are systematic reviews and metanalysis indicating that it does not differ from the general population, holding at 45% [101], and that it is significantly increased, reaching 63% [102]. There are also nationwide population studies indicating that OSA alone is a risk factor for developing PD at older age, such as analyses conducted in Korea [103] and Taiwan [104] on large groups of PD patients.

With regard to dopaminergic treatment, specifically dopamine agonists, it appears to have an ambiguous effect on SDB, as on the one hand it may contribute to the severity of central SDB, and on the other hand it reduced the severity of REM-related apnea/hypopnea index (AHI) in OSA suffering PD patients [105]. Later, it was shown that long-acting levodopa administered to patients at bedtime substantially reduced AHI [81,106]. This is consistent with previous studies that have shown levodopa to improve upper airway obstruction [107,108]. The effect of dopaminergic therapy on AHI may be indirect and related to improved motor activity at night, which allows patients to reduce the supine position during sleep, promoting upper airway collapse [109].

It should be noted that, in addition to the beneficial effects of L-DOPA on OSA, its adverse effects on breathing have also been previously reviewed and described, including diaphragmatic dyskinesia and short periods of apnea alternating with irregular rapid breathing, usually observed after reaching the maximum dose [110].

#### 3.2.3. Restless Legs Syndrome (RLS)

Restless legs syndrome (RLS) is a sensory-motor sleep disorder characterized by a wide range of sensory symptoms and unpleasant sensations affecting the lower limbs and causing the need to move the legs, as movement immediately relieves symptoms. RLS is more common in PD and is associated with severe motor and non-motor symptoms [111]. Intrajejunal levodopa infusion has been reported to significantly improve symptoms of nighttime restless legs in an open-label, prospective, observational, 6-month, multicenter study [93]. DA agonists may also be useful in treating RLS symptoms, but it is important to select the lowest effective dose to avoid exacerbating symptoms or other adverse effects such as hallucinations, withdrawal syndrome, and impulse control disorders [112]. It has been proven by numerous studies that dopaminergic agonists such as apomorphine, rotigotine, pramipexole, and ropinirole can be helpful in alleviating RLS [93,113,114,115,116,117,118,119]. Amantadine, which has both anti-glutamate and dopaminergic effects, also alleviated RLS symptoms comparable to ropinirole in a randomized open-label study, although it was less well-tolerated than ropinirole [118].

### 3.3. Pain

Pain is a frequent non-motor and heterogenous symptom in Parkinson’s disease. One of the factors contributing to prevalence of pain in PD is alterations in the dopaminergic system, which is considered to be involved in pain modulation, possibly through various cortical connections with the basal ganglia, including the limbic system and sensory cortex [120]. Approximately 40% of patients suffer from ‘primary pain’ in the early stages of the disease, before motor symptoms manifest. However, most PD-associated pain is secondary to motor impairment, i.e., stiffness, postural disorders, and motor fluctuations contributing to musculoskeletal, dystonic, and neuropathic pain [121,122]. According to a study by Vila-Cha et al. [123], nearly three-quarters of PD patients reported pain that was associated with more severe motor symptoms, anxiety symptoms, and comorbidities. Patients experienced pain that was classified as musculoskeletal (63%), dystonia-related (27%), centrally parkinsonian (22%), and/or radicular or neuropathic (9%). The causes of such a wide spectrum of pain syndromes in Parkinson’s disease are complex, and among the various factors, non-motor symptoms such as dysautonomia, sleep disorders, and mood disorders may also have an impact [122,124].

It has been suggested that levodopa may be effective in treating generalized non-specific pain in Parkinson’s disease, but it has little effect on nociceptive pathways and its pain-reducing effect is achieved mainly by improving the motor symptoms of PD patients. [125].

Nevertheless, according to a meta-analysis of the literature on hyperalgesia in Parkinson’s disease, dopaminergic medication consisting of levodopa, usually combined with DA agonists, was potent to reduce pain sensitivity [126]. In addition, PD patients showed a good response to levodopa treatment for musculoskeletal pain [127] and neuropathic, nociplastic, or nociceptive pain, especially after the drug wore off [128]. The addition of the DA agonist rotigotine in the form of a transdermal patch to the treatment of patients suffering from chronic pain, who were already receiving levodopa, alleviated chronic pain associated with advanced stage of the disease [129].

MAO-B inhibitors used as an adjunct to levodopa also exhibit pain-relieving effects. Numerous recent studies point to the beneficial effects of safinamide in particular. Oral safinamide at a dose of 100 mg per day relieved pain based on the PDQ-39 subscale for physical discomfort [130]. The effectiveness of this dose has been corroborated by open-label observational study conducted in Japan, which showed that safinamide relieved pain during OFF periods and at night [131]. A post hoc analysis of multicenter randomized control trial (RCT) studies involving patients with advanced PD demonstrated the long-term efficacy of safinamide (after two years) in the treatment of chronic pain. The addition of safinamide to a stable dose of levodopa was linked to a significant reduction in associated pain medication and amelioration in PDQ-39 bodily discomfort scale and musculoskeletal and neuropathic pain scores [132]. Another study, prospective open-label, conducted in five centers from Spain, showed that 6 months of safinamide treatment improved musculoskeletal, fluctuation-related, radicular, and nocturnal pain according to King’s Parkinson’s disease pain scale (KPPS) [133]. A systematic review analyzing 60 articles on MAO inhibitors showed that the effect of rasagiline on pain was inconsistent across the studies, which had either a beneficial effect on pain reduction or it had no effect at all [95]. Significant reduction in bodily discomfort domain was shown in Chinese [134] and Japanese [135] RCTs at a dose of 1 mg of rasagiline. Although rasagiline is considered a well-tolerated and effective drug, both in the treatment of early-stage Parkinson’s disease and as an adjunct to dopaminergic therapy in patients with PD and motor fluctuations, after discontinuation, in certain cases it may cause serious withdrawal syndrome characterized by psychiatric disorders and generalized pain [136]. Therefore, given the discrepancies regarding the effect of rasagiline on pain and the adverse effects following discontinuation, it does not appear to be clinically relevant for the treatment of pain.

In the case of COMT inhibitors, no pain-relieving effect was observed; moreover, in older studies some patients experienced abdominal pain as side effects associated with taking entacapone or tolcapone [137].

There is not much evidence that amantadine can affect pain in PD. In 2006 [138] it was demonstrated that amantadine sulfate reduced experimental hypersensitivity and pain perception in patients with chronic back pain, suggesting its potential in the treatment of chronic pain in PD. Post hoc analysis of clinical trials of extended-release amantadine revealed a sustained reduction in painful dystonia during the OFF phase in patients with PD, regardless of the reduction in OFF phase duration [139]. A recent promising cross-sectional study of PD patients assessing the prevalence and characteristics of pain showed that those taking amantadine presented less radicular/neuropathic pain [140].

### 3.4. Neuropsychiatric Manifestations in Parkinson’s Disease

With the ongoing progression of Parkinson’s disease, the number of patients experiencing certain neuropsychiatric symptoms (NPS) increases, including mood disorders, fatigue, psychosis, cognitive impairment, and addictions [141,142]. According to systematic review of 30 studies, the most frequent NPSs were mood disorders: depression (47%), apathy (45%), and anxiety (42%). Psychosis had an average prevalence of 19% and impulse control disorders (ICD) of 18% [142]. In some patients with moderate-to-severe Parkinson’s disease, anxiety or dysphoric mood is associated with the occurrence of motor fluctuations as the effects of the last dose of medication wear off, and with dyskinesias when the dose of dopaminergic medication is too high or as a side effect of long-term levodopa treatment [143]. Therefore, individual optimization of dopaminergic therapy is recommended for all PD patients, as it can significantly alleviate non-motor symptoms associated with ‘off periods’, including anxiety and depression [144,145]. Levodopa therapy has been described to improve the hallmark of apathy, i.e., reduced cognitive motivation, to such an extent that the choices made by patients taking the medication were indistinguishable from those of the control group [146]. Levodopa has been reported to alleviate the psychological symptoms of PD more effectively than dopamine agonists improving not only depression, as in case of DA agonists, but also anxiety, somatization, and global psychiatric index [147,148]. Additionally, the use of dopaminergic agonists may be associated with the onset of ICD [143]. A longitudinal study analyzing data from a multicenter cohort of PD patients who frequently took DA agonists showed that the incidence of ICD increased significantly after 5 years of treatment and gradually decreased after discontinuation of therapy [149]. Nevertheless DA agonists have been shown to have beneficial effects on other neuropsychiatric symptoms. Two meta-analyses of randomized clinical trials showed that the dopaminergic agonist rotigotine, administered in the form of a transdermal patch, significantly alleviated apathy and depression [150,151]. A prospective cross-sectional study of PD patients without dementia treated with pramipexole or ropinirole, either as monotherapy or with levodopa showed that the use of pramipexole was linked with a lower frequency and severity of apathy symptoms [152]. The positive effect of pramipexole has been described later on anhedonia [153], anxiety, and depression [127,154] in PD patients. Stimulation of D_2_/D_3_ receptors with another DA agonist, piribedil, effectively alleviated symptoms of depression and apathy, as demonstrated in a 12-week prospective, placebo-controlled, randomized, double-blind trial involving 37 patients with PD [155]. In turn, the MAO-B inhibitor safinamide showed benefits in terms of ‘emotional well-being’, significantly improving the PDQ-39 score after six months and two years of treatment, while also reducing the experience of depression [156]. A subsequent meta-analysis of RCT investigating the effects of three MAO-B inhibitors, selegiline, rasagiline, and safinamide, revealed their efficacy in reducing symptoms of depression, particularly in patients with early-stage Parkinson’s disease [157]. Among COMT inhibitors, opicapone has been shown to be effective after 6 months of treatment in reducing mood changes and apathy [158,159], as well as anxiety/panic attacks [159]. A study analyzing three phase III clinical trials involving 6 months of treatment with amantadine in the form of extended-release capsules showed its effectiveness in reducing symptoms of depression. At the same time, adverse effects of amantadine were observed in the form of an increased incidence of hallucinations/psychosis, its well-known side effect [99]. Amantadine also showed other beneficial effects; in adjunctive therapy, it reduced reward hypersensitivity and risky choices and increased non-risky choices in PD patients with severe pathological gambling [160]. However, this positive effect on ICD was more likely to be due to its anti-glutamatergic action than to its activation of the dopaminergic system. There are also reports of the positive effects of amantadine on cognitive impairment in patients with Parkinson’s disease. A study conducted on a small group of subjects showed that six months of treatment with amantadine sulfate yielded a significant improvement in neuropsychological test scores [161]. The latest summary of two clinical trials, which also involved a small group of patients, showed improvement in verbal and auditory memory and visuospatial processing in PD patients, but only in those treated with amantadine monotherapy for six months and one year [162]. As these are small-scale studies, further clinical trials involving large populations of patients are needed to confirm the beneficial effect of amantadine on cognitive function in patients with PD.

**Figure 3:**
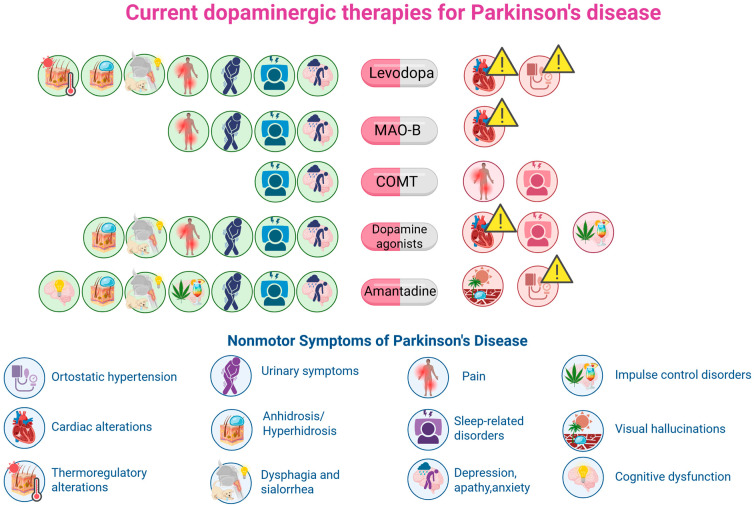
Non-motor symptoms that may be reduced or/and exacerbated by dopaminergic treatment in Parkinson’s disease. The green symbols to the left of the medications indicate a beneficial effect on symptoms, while the red symbols to the right indicate an exacerbation of symptoms.

A summary of the effects of dopaminergic therapies on non-motor symptoms described in this review is presented in Figure 3 and Table 1.

## 4. Conclusions

Parkinson’s disease is a complex neurodegenerative disorder characterized by clinical symptoms that spread well beyond the classic motor phenotype increasing the disease burden, speeding up disability, and profoundly affecting quality of life. A number of the non-motor symptoms of PD described and synthetized in this review are associated with dysfunction of the dopaminergic system such as thermoregulatory dysfunction, urinary symptoms, or sleep-related disorders. Accordingly, certain symptoms, although deemed invulnerable to dopaminergic drugs, may be mitigated by targeted and individualized dopaminergic therapy.

The limitations of our review reside in the fact that it focuses on scientific reports from recent years, often omitting older works that have been described previously, and that it does not assess the reliability of the research, especially if the outcomes were consistent. However, in cases where the research results were contradictory, we attempted to assess the quality of the research in terms of sample size or the usage of RCTs or open-label studies. It was also challenging to draw conclusions on the beneficial and/or undesirable effects of specific dopaminergic treatments due to inconsistencies in research data and medical protocols, as well as incomplete information. Sometimes there is very little or no research on a specific symptom or therapy. As in the case of amantadine, which has a beneficial effect on many non-motor symptoms, however, this is confirmed by a single study. The picture that emerges from this review certainly allows us to conclude that the vast majority of dopaminergic therapies, with the exception of COMT inhibitors, have an unfavorable profile in terms of inducing or exacerbating orthostatic hypotension or adverse cardiac changes. Regarding beneficial effects, most of these therapies improve urinary tract function, sleep disorders, depression, apathy, and anxiety. Of course, there are exceptions, such as the COMT inhibitor, whose addition worsened sleep quality, and certain agonists that caused daytime sleepiness. The benefits in reducing pain have been confirmed for levodopa, MAO-B, and dopamine agonists, while levodopa, agonists, and amantadine have been shown to reduce dysphagia/sialorrhea and anhidrosis/hyperhidrosis. It is necessary to be cautious in the dosage and selection of therapy, as dopaminergic drugs may have a positive effect on some non-motor symptoms while exacerbating others. An example of this is the beneficial effect of DA agonists in the treatment of pain or RLS, with the simultaneous risk of ICDs. It is of note that the sudden discontinuation of therapy may also develop acute withdrawal syndrome characterized by psychiatric disorders, generalized pain, hyperhidrosis, or hyperthermia.

In summary, the treatment of Parkinson’s disease with dopaminergic drugs should pursue a precise and personalized therapy that goes well beyond motor phenotypes and takes into account non-motor areas, starting from the prodromal stage. To establish effective therapies based on dopaminergic activation, solid clinical trials focusing on the use of specific drugs in the treatment of non-motor symptoms, especially those underrepresented, are needed.

## Figures and Tables

**Table 1:** Non-motor symptoms of Parkinson’s disease and their responsiveness to dopaminergic therapies.

	Levodopa	MAO Inhibitors	COMT Inhibitors	DA Agonists	Amantadine
Autonomic Dysfunction in Parkinson’s Disease					
Orthostatic Hypotension	− [28,29,30,31]				− [22]
Cardiac Alterations	− [34,35]	− [37]		− [38]	
Thermoregulatory Alterations	+ [42]/− [46,47]				
Urinary Symptoms	+ [67,68]	+ [69,70,72]		+ [55,74]	+ [73]
Anhidrosis/Hyperhidrosis	+ [53]			+ [54]	+ [55]
Dysphagia and Sialorrhea	+ [58]			+ [61,62]	+ [63]
Sensory Alterations in Parkinson’s Disease					
Pain	+ [126]	+ [130,131]	− [137]	+ [126,129]	
Sleep-Related Disorders in Parkinson’s Disease					
REM Sleep Behavior Disorder	+ [86]				
Insomnia and Sleep Fragmentation	+ [85]	+ [95,96]	+ [88,89]	+ [55,90,91,94]	
Excessive Daytime Sleepiness (EDS)		+ [97]	− [98]	+ [91]/− [92]	+ [99]
Obstructive Sleep Apnea (OSA)	+ [107,108]/− [110]				
Restless Leg Syndrome (RLS)	+ [93]			+ [93,113,114,115,116,117,118,119]	+ [118]
Neuropsychiatric Manifestations in Parkinson’s Disease					
Depression	+ [147,148,155]	+ [156,157]		+ [150,151]	+ [99]
Apathy	+ [146,155]		+ [158,159]	+ [150,151,152]	
Anxiety	+ [147,148]		+ [159]	+ [154]	
Visual Hallucinations					− [99]
Impulse Control Disorders (ICD)				− [143]	+ [160]
Cognitive Dysfunction					+ [162]

+ means positive effect, − means negative effect, [] brackets contain literature reference showing the effect.

## Data Availability

No new data were created or analyzed in this study. Data sharing is not applicable to this article.
